# Therapeutic and Clinical Potential of Adipose-Derived Stem Cell Secretome for Skin Regeneration

**DOI:** 10.3390/cells14211727

**Published:** 2025-11-04

**Authors:** Anna Sendera, Hubert Kubis, Anna Pałka, Agnieszka Banaś-Ząbczyk

**Affiliations:** 1 Department of Medical Biology, Faculty of Medicine, Collegium Medicum, University of Rzeszów, al. Tadeusza Rejtana 16C, 35-959 Rzeszów, Poland; anpalka@ur.edu.pl; 2 Laboratory of Diagnostic and Clinical Epigenetics, Faculty of Medicine, Collegium Medicum, University of Rzeszów, al. Tadeusza Rejtana 16C, 35-959 Rzeszów, Poland

**Keywords:** stem cell therapy, adipose-derived stem cells, extracellular vesicles, exosomes, conditioned media, AT-MSCs, skin aging prevention, wound healing, alopecia treatment, preconditioning

## Abstract

Adipose-derived stem cells (AT-MSCs) exhibit great potential for application in stem cell therapy, primarily due to their unique pro-regenerative capabilities, which include supporting skin regeneration. AT-MSCs secrete a variety of biomolecules with immunomodulatory, re-epithelializing, antifibrotic, antiapoptotic, proangiogenic, and neurotrophic activity as well as the ability to promote proliferation and migration of skin cells. Recently, therapy using AT-MSC secretome alone has garnered increasing attention due to its potentially safer and more effective application ability than the use of whole AT-MSCs. This review provides a comprehensive summary of the current state of knowledge regarding the potential use of AT-MSC secretome as a promising cell-free therapeutic strategy for wound healing, alopecia, skin rejuvenation, and skin inflammatory diseases. We critically analyze and discuss findings from *in vitro*, *in vivo*, and clinical studies. Moreover, we briefly discuss possible approaches to enhance the secretion of AT-MSC biomolecules, such as AT-MSC preconditioning with low-frequency electromagnetic fields and hypoxia. In conclusion, the evidence presented in this review paper underscores that the AT-MSC secretome alone can be a highly effective approach as a stem cell-free therapy for skin repair, with significant translational potential.

## 1. Introduction

The skin, the largest organ of the human body, is constantly exposed to external factors and performs protective, defensive, thermoregulatory, and sensory functions [1]. The skin is a complex, multilayered organ comprising the epidermis, dermis, and subcutaneous layer from the surface inward. The epidermis consists of several cellular strata, including the stratum corneum, stratum lucidum, stratum granulosum, stratum spinosum, and stratum basale. Beneath the epidermis lies the dermis, which is rich in collagen and elastin fibers, as well as an extensive network of blood vessels and nerve endings [2]. The deepest structure is the subcutaneous layer, which is composed primarily of adipose tissue (AT). Exposure to solar radiation, pollutants, hyperglycemia, and other internal and external factors may adversely affect skin condition and functional properties, including its impaired regeneration. Therefore, maintaining healthy skin and supporting its regeneration are essential for ensuring its proper functioning and effective protection of the body [3,4,5,6].

Mesenchymal stem cells (MSCs) have great potential for use in stem cell therapies. They can be isolated from various human tissues, but most commonly, bone marrow, adipose tissue, and umbilical cord (Wharton’s jelly, cord blood) are considered as sources for use in regenerative medicine [7,8]. According to the clinical experience of Hoang et al. [8], MSCs derived from AT are highly promising candidates for therapeutic applications targeting skin-related conditions owing to their unique secretome composition, which supports skin regeneration processes. In the context of cell-based therapies, the secretome comprises a spectrum of cell-derived components, including exosomes and extracellular vesicles, while conditioned medium represents a complex mixture of soluble proteins, cytokines, growth factors, and vesicular elements such as exosomes and extracellular vesicles [9]. The secretome of adipose-derived stem cells (AT-MSCs) consists of many biomolecules with immunomodulatory, re-epithelializing, anti-fibrotic, antiapoptotic, proangiogenic, and neurotrophic properties, as well as the ability to support the growth and migration of skin cells. Therefore, it can support wound healing, psoriasis management, skin rejuvenation, or hair regrowth [7,10,11,12].

New therapeutic methods for skin regeneration encompass a wide range of clinical applications, including wound healing, skin-aging, alopecia, and immune-related skin disorders, and have attracted a lot of interest. While wound healing remains a critical focus particularly for chronic and hard-to-heal wounds, advancements in skin aesthetics, structural tissue reconstruction, and specialized skin structures such as hair follicle regeneration have expanded the scope of skin regeneration therapies [10,13,14]. Skin regeneration involves complex molecular and biochemical mechanisms, which vary depending on the specific disorder being addressed [10,15,16,17,18]. For instance, the wound healing process progresses through inflammation, debridement, repair, and maturation [10,15]. In contrast, skin aging treatments focus on collagen stimulation and improving skin elasticity, [19,20], while approaches for immune-related skin disorder treatments aim to modulate the immune response and reduce inflammation [21,22]. In turn, alopecia therapies target hair follicle regeneration [16]. The success of skin regeneration therapies depends on understanding the pathophysiological mechanism underlying the disease, which varies and thus requires tailored therapeutic approaches [20].

As numerous researchers indicate that stem cells—particularly those derived from adipose tissue—play a crucial role in skin regeneration and can be successfully used in applications for skin-related conditions [8,12,23,24,25,26,27,28]. AT-MSCs may be applied in both stem cell-based and stem cell-free therapeutic approaches. However, increasing evidence has shown that the regenerative properties of MSCs are based on their secretome activity (paracrine mechanism) rather than their differentiation ability (reconstructive mechanism). Therefore, stem cell-free therapy, a next-generation therapeutic strategy that uses only the MSC secretome (exosomes, extracellular vesicles, or conditioned medium), may constitute a more effective and safer therapeutic approach for skin regeneration application [7,8,9,29,30].

In this review, we collected the current state of knowledge regarding the potential of adipose-derived stem cells’ secretome for skin regeneration applications. Special attention was given to the paracrine effect of the AT-MSC secretome in wound healing, alopecia, skin rejuvenation, and skin inflammatory diseases. The review provides evidence from *in vitro*, *in vivo*, and clinical studies involving the use of the AT-MSC secretome for skin regeneration to highlight its therapeutic potential and future perspectives for stem cell-free therapies in dermatology.

## 2. Adipose-Derived Stem Cell Characterization

Adipose-derived stem cells are a type of mesenchymal stem cell originating from adipose tissue, characterized by their capacity for differentiation. AT-MSCs share characteristics with MSCs, according to the criteria established in 2006 by the International Federation for Adipose Therapeutics and Science (IFATS) and the International Society for Cellular Therapy (ISCT). These criteria include fibroblast-like morphology, the ability to adhere to plastic surfaces *in vitro*, expression of surface markers such as CD73, CD90, and CD105, absence of expression of markers CD11b, CD14, CD19, CD45, and HLA-DR, as well as the potential to differentiate into three cell lineages: osteogenic, chondrogenic, and adipogenic [7,31]. However, the latest ISCT recommendations for MSC characterization were updated in 2025. The recommendations from 2006 need to be revised, as proposed surface markers for 2D culture have been proven insufficient for reliable identification of MSCs derived from different sources (tissues) and cultured under diverse protocols. ISCT advises that researchers should mention the tissue of origin as a prefix when naming MSCs; therefore, in this work, instead of the acronym for those cells (ASC) commonly used by authors in previous works, to provide greater clarity of tissue origin, the term AT-MSC has been used. The updated recommendations clearly emphasize that tissue origin significantly influences MSC surface marker profile and functional properties, highlighting the importance of adopting more precise and source-dependent criteria for MSC characterization [32]. AT-MSCs originating from different anatomical sites or species may exhibit variations in density. Furthermore, the selection of an appropriate isolation method can influence cell quality, plasticity, and functionality [33,34]. The most abundant source of AT-MSCs is subcutaneous AT [35]. AT-MSCs isolated from white adipose tissue (WAT) exhibit different characteristics than those derived from brown adipose tissue (BAT), reflecting the distinct functions of these fat depots. BAT primarily functions in the maintenance of body temperature. Adipocytes within BAT are multilocular and contain smaller lipid vacuoles compared to adipocytes from WAT, which is distributed throughout the body and serves as a lipid storage reservoir [36].

In 2001, Zuk et al. developed a widely adopted method for isolating AT-MSCs from WAT [37] that has undergone numerous modifications [38,39]. These primarily include various AT isolation techniques, such as surgical resection, power-assisted liposuction (PAL), and laser-assisted liposuction [34], the removal of erythrocytes using a NaCl solution [40], free stratification, and centrifugation under varying centrifugal forces [38]. A limited number of publications comparing the efficiency of these modified methods precludes establishing a single validated procedure for AT-MSC isolation. Adipose tissue is typically harvested via liposuction and, according to the original protocol by Zuk et al., washed with physiological saline solution, minced into small fragments, and then subjected to type II collagenase digestion at 37 °C for 30 min. The enzyme is inactivated using Dulbecco’s Modified Eagle Medium containing 10% fetal bovine serum. This process yields the stromal vascular fraction (SVF), which includes AT-MSCs, preadipocytes, lymphocytes, leukocytes, and erythrocytes. The cell pellet is incubated with NH_4_Cl to lyse erythrocytes. Residual undigested tissue components are removed by filtration through a 100 μm sieve, and the cells are cultured on a growth medium as an adherent culture [37].

Current therapeutic approaches in skin regeneration include the use of extracellular matrix (ECM) derived from AT-MSCs, direct application of AT-MSCs, the SVF containing AT-MSCs, and the secretome secreted by AT-MSCs [41,42,43,44]. AT-MSCs have the capacity to produce numerous compounds that constitute the secretome. This secretome comprises cytokines, growth factors, morphogens, and chemokines, as well as extracellular vesicles (EVs), including exosomes and ectosomes. Importantly, EVs carry non-coding RNAs (e.g., miRNA) that modulate gene expression, thereby supporting the regenerative process and tissue regeneration. Extracellular vesicles, ectosomes, and exosome structures differ in their mechanisms and sites of biogenesis, as well as their content. EVs are lipid bilayer-enclosed vesicles formed either by outward budding from the plasma membrane or from the internal endosomes. Exosomes originate from multivesicular bodies within the endocytic pathway, whereas ectosomes are directly shed from the plasma membrane [45,46,47]. These mediators are essential for the proper progression of wound healing, as they regulate cell proliferation and migration, angiogenesis, immune response, and inflammatory mechanisms [48,49,50,51,52]. The secretome derived from AT-MSCs, as a cell-free product, is considered a safer therapeutic alternative compared to therapies based on the direct application of AT-MSCs [7,8,9,30].

## 3. Stem Cell Therapy and Stem Cell-Free Therapy Approaches

**Figure 1:**
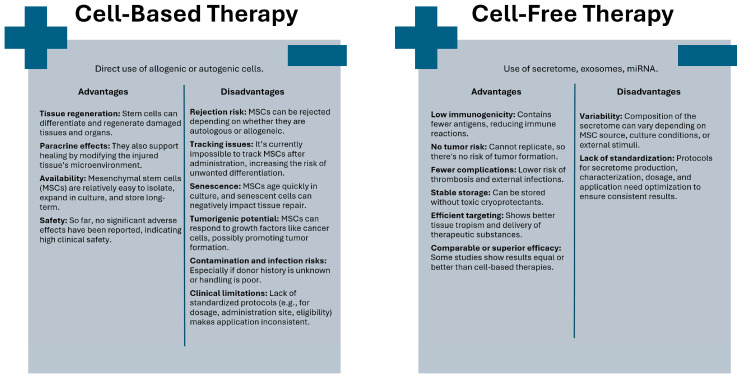
Comparison of cell-based and cell-free therapeutic approaches.

From a clinical perspective, stem cells can be utilized either directly in stem cell-based therapy or indirectly through the molecules they produce in stem cell-free therapy [13,53,54] (Figure 1).

Cell-based therapies are defined as the therapeutic or medicinal use of any type of living human stem cell, most commonly administered via injection into the target tissue or organ [55]. The direct use of stem cells represents an ideal solution for tissue and organ transplantation because, due to their differentiation capacity, stem cells can facilitate the repair and regeneration of damaged or diseased tissue. In addition to tissue and organ reconstruction, paracrine activity and modification of the microenvironment of injured tissues ultimately lead to their renewal [56]. At this stage, selecting the appropriate type of mesenchymal stem cell is essential, depending on clinical application [8,57]. Despite this, MSCs are relatively easy to isolate and expand in cell culture, and can be banked long-term without loss of function. Moreover, no significant adverse effects associated with their use have been reported, which is why they are considered a clinically safe therapeutic approach [58].

However, the direct application of stem cells in therapies has its limitations. Sreenivas et al. identified four categories highlighting the constraints associated with the use of MSCs in treatment. The first category, intrinsic factors, pertains to the rejection of transplanted MSCs due to their origin, whether autologous or allogeneic. The next category concerns the inherent characteristics of MSCs themselves [59]. Currently, it remains impossible to track the fate of MSCs in the recipient, which may increase the risk of undesirable cell differentiation and lead to rejection of these cells [60]. Some applications require achieving a specific number of MSCs, while MSCs cultured *in vitro* undergo rapid senescence. Moreover, during the aging process, MSCs exhibit morphological, genetic, and phenotypic changes [61]. The introduction of senescent MSCs into the recipient’s tissue or organ may lead to the spread of various senescence-associated secretory phenotype (SASP) factors among the cells, which can inhibit tissue regeneration and affect neighboring cells [61,62]. MSCs also possess tumorigenic potential, as they respond to growth factors similar to those affecting cancer cells, which may promote tumor development [63]. The next category of limitations concerns external factors, such as infections resulting from a lack of donor health history, the risk of contamination due to inadequate cell handling procedures, and those related to chemicals used during cell culture. Additionally, this category includes clinical considerations, where Sreenivas et al. highlighted adverse immune responses, reactions at the injection site, absence of a defined administration site, and lack of standardized criteria for therapy eligibility, optimal dosage, and injection timing [59].

The literature emphasizes the necessity of developing alternative methods for the specific isolation and precise characterization of MSCs due to their high heterogeneity and challenges in their unequivocal definition. Additionally, there remains a lack of consensus regarding the optimal ex vivo culture protocol, purity and sterility standards, a uniform assay for cell identity verification, the most effective administration route, appropriate dosing, and the frequency of cell application [64,65,66].

Due to the challenges and limitations associated with the clinical application of MSCs, cell-free therapies have garnered much greater interest. Furthermore, numerous studies report that the therapeutic effects are primarily attributed to MSC-derived products rather than the transplanted MSCs themselves [9,11,67]. MSC-derived products are utilized to regulate numerous processes, including apoptosis, cell proliferation, migration to the injury site, and angiogenesis [68]. Stem cell-free therapies can thus be recognized as a next-generation therapeutic strategy [11,13,30]. The cell-free form administered intravenously is associated with a lower risk of thromboembolic complications. Inability to replicate can eliminate the risk of tumorigenic transformation. Due to its limited antigen content, this preparation is weakly immunogenic. Additionally, it reduces the risk of external infections, which can be observed in therapies based on live cells. It can be stably stored without the need for toxic cryoprotectants. Moreover, it demonstrates improved tropism for target tissues, facilitating effective delivery of active substances. Reports indicate that its efficacy may be comparable to or even superior to that of conventional cell-based approaches [13,53,69,70,71]. The complete cellular secretome (in the form of conditioned medium) and extracellular vesicles such as exosomes are commonly selected for cell-free MSC applications, as described, among others, by Tan Mi et al. [53]. However, it is important to note that the composition of the MSC-derived secretome may vary depending on MSC tissue origin, cell isolation method, culture conditions, or external stimuli with physical or biochemical factors. Therefore, the standardization and optimization of protocols of secretome use in different clinical applications are crucial to maximize its therapeutic potential and ensure consistent clinical outcomes [9,54].

### Isolation and Preparation Methods for MSC-Derived Products: Challenges and Perspectives

To effectively utilize secretomes derived from AT-MSCs, standardization of research methods is necessary due to numerous confounding factors such as donor sex, age, body mass, secretome collection method, and the anatomical area from which the AT-MSCs are obtained. Regarding the secretome collection method, factors important from the perspective of which proteins are detected and in what quantities include the type of culture medium used, culture conditions (e.g., 2D and 3D culture; oxygen level; biochemical stimuli), differences in cell incubation times, the method of secretome purification and concentration, and the presence or absence of serum. The standardization of AT-MSC secretome harvesting is essential since it directly influence its effectiveness [29,72]. One of the most important factors influencing AT-MSC secretome composition is culture conditions. For example, currently, the standard model for cell culture and expansion is 2D culture; however, 3D culture better reflects the physiological environment of stem cell niche. Hodge et al. [73] showed that 3D culture can increase the secretion of pro-regenerative factors, including those such as EV in conditioned media. It influenced improved proliferation and migration of keratinocytes and fibroblasts treated with secretome obtained from 3D culture compared to secretome from 2D culture [73]. The same research group 2 years later compared three different 3D cell culture models. They again showed that a 3D hydrogel culture system can significantly enhance secretion of secretome proteins such as IL-10 or CSF2 and EV concentration. However, it is important to note here that depending on the type of 3D technique (spheroids/Matrigel/Bio-Blocks), the effect on AT-MSC secretome composition was different. It also influenced endothelial cells stimulated with AT-MSC extracellular vesicles (AT-MSC-EVs) differently—when Bio-Block-obtained EVs enhanced cell proliferation and migration, the spheroid EVs induced cell senescence and apoptosis [74]. Similarly, Al-Shaibani [75] showed increased concentrations of pro-regenerative proteins in AT-MSC conditioned medium (AT-MSC-CM) obtained from 3D culture (polystyrene scaffold) compared to 2D culture [75]. Therefore, a comprehensive understanding of the influence of culture conditions on the AT-MSC secretome represents a critical step toward the optimization of its therapeutic utility.

The protein detection method is also crucial because some proteins may be detected only in certain studies, which complicates comparisons. Therefore, it is important to consider the use of different technologies (e.g., mass spectrometry vs. ELISA), their selectivity toward specific protein classes, and the varying sensitivity and specificity of these methods. There is also a lack of uniform standards in reporting results, making it difficult to perform meta-analyses or identify common features of secretomes [29,72].

Giannasi et al. [76], also emphasized the necessity of a multi-step approach to secretome standardization. They stated that it is important to have an overview of the entire secretome composition, in this case using Raman spectroscopy and nanoparticle tracking analysis, as well as to perform quantitative measurement of molecules of various types [76]. However, techniques such as serial analysis of gene expression, DNA microarrays, antibody and bead-based methods, mass spectrometry, RNA sequencing, and bacterial/mammalian secretion traps also can be used for quantitative and qualitative secretome characterization [72]. Obtained secretome analysis is important because key components of the AT-MSC secretome may be responsible for its therapeutic effects [76]. For example, AT-MSCs have the potential to enhance graft regeneration and vascularization; however, studies show that the number of stem cells obtained depends on several factors, such as the tissue harvesting method (e.g., liposuction), cell isolation procedures, and cell counting techniques (counting chamber, colony counting, flow cytometry). Even when tissue is harvested from the same site in the same patient, the results can vary significantly depending on the isolation method and the quantification technique used. In study [77], where stem cells were isolated after liposuction and quantification was performed using a counting chamber or flow cytometry, it was confirmed that from a clinical perspective, there is a great need to optimize stem cell yield using non-enzymatic methods during lipotransfer [77].

Understanding how processing techniques influence the regenerative capacity of AT-MSC secretome is crucial for their effective clinical use. Prantl et al. [78], examined how the method of mechanical processing of adipose tissue (lipoaspirates) affects the quality of stem cells and their secretome. They investigated whether mechanical processing, such as centrifugation and homogenization, allows enrichment of samples with stem cells without altering their secretory properties. Adipose tissue was harvested from patients using water jet–assisted liposuction. The samples were then subjected to double centrifugation and homogenization by passing between syringes. The number of stem cells and the composition of secreted proteins (secretome) were analyzed using mass spectrometry, which confirmed that the secretome composition did not change significantly, while mechanical processing increased the number of stem cells by an average of 2.6-fold. This is significant from the perspective of regenerative medicine and aesthetic surgery [78].

An equally important aspect is the preparation of exosomes and their potential applications in therapy. Several isolation and characterization methods can be distinguished that allow differentiation of extracellular vesicle subgroups, such as size exclusion or affinity chromatography, filtration, ultracentrifugation, reactive ligand technology, and antibody technology. In article [79], the authors describe the potential use of exosomes derived from AT-MSCs in the therapy of triple-negative breast cancer; however, the research is currently at the *in vitro* stage [79].

However, it is important to note here that selection of the type of AT-MSCs-derived product either in the form of conditioned medium, extracellular vesicles, or exosomes may have different therapeutic effects. For example, Giannasi et al. showed that AT-MSC-CMs have higher therapeutic potential compared to AT-MSC-EVs alone for an osteoarthritis model *in vitro* [80]. Nevertheless, it should be noted that the therapeutic response may also vary depending on the underlying disease and experimental model used in experimental study (*in vitro*/*in vivo*) [81].

## 4. Adipose-Derived Stem Cell Secretome in Skin Regeneration

### 4.1. Chronic Wounds

From a dermatological perspective, one of the greatest challenges in skin regeneration is the management of chronic wounds. It is estimated that 1–2% of the global population suffers from hard-to-heal wounds caused by various factors [14]. These factors can be categorized into chronic diseases such as diabetes, obesity, chronic venous insufficiency, arterial disease, and diabetic foot syndrome. Additionally, pressure ulcers, infections—particularly those caused by Gram-negative bacteria forming biofilms—metabolic disorders like vitamin D deficiency, as well as external factors including trauma, surgeries, and accidents, can all influence the rate of wound healing [14,82,83].

The secretome of AT-MSCs represents a promising therapeutic alternative for the treatment of chronic wounds, particularly diabetic foot ulcers, aiming to address the challenges these wounds pose and improve patient outcomes (Table 1).

#### 4.1.1. AT-MSC Secretome in Diabetic Chronic Wound Healing

Diabetic ulcers arise as a result of impaired self-healing capacity, reduced tissue regenerative ability, and damage to blood vessels and nerves caused by hyperglycemia in diabetes [5,6,84]. Excessive hyperglycemia contributes to the development of oxidative stress, activation of inflammatory processes, and skin dysfunction, which ultimately leads to the formation of refractory diabetic wounds [85]. Current treatment methods for diabetic wounds focus on the use of antibiotics, antimicrobial agents, and wound care. However, their effectiveness is significantly limited [86,87].

Scientists have demonstrated that AT-MSC exosomes can enhance the promotion of vascular regeneration, which is essential for wound healing due to the transport of nutrients and oxygen to the wound site. Zhang et al. showed that AT-MSC exosomes can mitigate oxidative stress and reduce the secretion of proinflammatory cytokines in diabetic wounds, thereby promoting angiogenesis [88]. Furthermore, Ge et al. obtained modified AT-MSC exosomes overexpressing miR-132, and their application in diabetic wounds rapidly stimulated the formation of a greater number of new blood vessels compared to the control group [89].

Moreover, Hsu et al. demonstrated that treatment with AT-MSC exosomes resulted in enhanced proliferation of dermal fibroblasts and keratinocytes, improved re-epithelialization, wound contraction, and angiogenesis compared to cells derived from diabetic dermal fibroblasts. This effect was mediated through the stimulation of monocytes/macrophages to secrete increased levels of TGF-β1, which is responsible for fibroblast activation [90]. Conversely, Liang et al. demonstrated that AT-MSC exosomes enhance fibroblast proliferation and migration while decreasing the expression of TGF-β1 and alpha-smooth muscle actin (α-SMA). This effect is attributed to the elevated levels of miR-128-1-5p, which inhibits TGF-β1. Such modulation contributes to the promotion of diabetic wound healing and the suppression of skin fibrosis and scar formation through regulation of the miR-128-1-5p/TGF-β1/Smad signaling pathway [91].

In addition, Qiu et al. demonstrated that AT-MSC exosomes overexpressing the long non-coding RNA linc00511 promote the healing of diabetic foot ulcers and stimulate angiogenesis by inhibiting the ubiquitin-mediated degradation of Twist1, which is induced by Progestin and AdipoQ Receptor Family Member 3 (PAQR3) [92]. Another study by Liang et al. demonstrated that circular RNA mmu_circ_0001052 derived from AT-MSC exosomes promotes angiogenesis via the miR-106a-5p and FGF4/p38 MAPK signaling pathway [93].

The components of the AT-MSC secretome—neurotrophic factors such as basic fibroblast growth factor (bFGF/FGF-2), insulin-like growth factor 1 (IGF-1), nerve growth factor (NGF), and neurotrophins 3 and 4 (NT-3 and NT-4)—play a critical role in nerve regeneration [94]. Moreover, molecules including miRNA-130a-3p and miRNA-26b are involved in remyelination processes and stimulate the proliferation of Schwann cells, thereby inhibiting the progression of diabetic neuropathy [95,96]. Furthermore, components of AT-MSC-conditioned medium modulate the pro-inflammatory microenvironment, which promotes neurodegenerative processes during diabetes, shifting it towards an anti-inflammatory milieu that protects neuronal cells and supports their proliferation. The AT-MSC secretome inhibits neural autophagy and apoptosis while promoting remyelination, thereby creating regenerative conditions favorable for nerve repair [94,96].

Recently, evidence has increasingly highlighted the role of autophagy in the wound healing process [97,98,99]. The administration of AT-MSC exosomes has been shown to stimulate the autophagy, as evidenced by enhanced proliferation and migration of epidermal cells, thereby facilitating the healing of diabetic wounds. Ren et al. reported an upregulation of autophagy-related genes, such as nicotinamide (NAMPT), CD46, vesicle-associated membrane protein 7 (VAMP7), VAMP3, and the alpha subunit of eukaryotic translation initiation factor 2 (EIF2S1), following treatment with AT-MSC exosomes in diabetic wounds in mice. These findings suggest a potential mechanism of action for AT-MSC exosomes in the context of diabetic wound healing [100].

Additionally, Song et al. demonstrated that AT-MSC-derived exosomes not only promote the proliferation and migration of rat fibroblasts but also mitigate myofibroblast differentiation and collagen deposition, leading to scarless healing of diabetic wounds. These effects are potentially mediated by miR-204-5p present in the exosomes, which inhibits the TGF-β1-induced p-Smad2/3 pathway, as well as Col I and α-SMA expression [28].

Interestingly, Song et al. demonstrated a novel technology highlighting the potential application of ECM hydrogels combined with AT-MSC exosomes from healthy C57BL/6 mice. This approach enabled sustained, localized release of exosomes from the ECM hydrogel at the wound site. Both *in vivo* and *in vitro* studies showed that the therapy reduced inflammation, promoted angiogenesis, enhanced collagen deposition, and stimulated cell proliferation and migration, ultimately accelerating the healing of both diabetic and normal wounds. This innovative strategy suggests a promising new direction for wound healing. However, as noted by Song et al., further detailed investigations are required, particularly to identify and characterize the specific components of exosomes and their potential roles in skin regeneration and wound repair [24].

#### 4.1.2. AT-MSC Secretome in Non-Diabetic Chronic Wound Healing

Hodge et al. demonstrated that culturing AT-MSCs within a 3D tissue-mimetic hydrogel system markedly enhances their secretory activity compared with conventional 2D culture. AT-MSCs maintained in a 3D microenvironment preserved higher expression of stemness markers, exhibited reduced cellular senescence, and showed increased secretion of protein factors, antioxidants, and extracellular vesicles. Conditioned medium obtained from the 3D culture significantly enhanced the metabolic, proliferative, and migratory activity of keratinocytes and fibroblasts—two key cell types involved in wound repair—highlighting the regenerative potential of the AT-MSC secretome produced under biomimetic conditions [73].

The study by Carter et al. demonstrated that the use of 3D electrospun scaffolds as a culture environment for MSCs significantly enhances their paracrine potential compared with conventional 2D culture. Unlike the work of Hodge et al., which examined the general secretory properties of AT-MSCs, Carter and colleagues focused specifically on corneal wound healing. The secretome of MSCs cultured in the 3D system contained markedly higher levels of factors such as HGF and ICAM-1, resulting in faster proliferation and migration of corneal fibroblasts, as well as improved epithelialization and reduced scarring in an ex vivo corneal model. Immunostaining revealed decreased α-SMA expression, indicating inhibition of myofibroblast transformation and attenuation of fibrosis [101].

From a wound-healing perspective, this study underscores that optimization of the MSCs’ culture microenvironment can profoundly influence their phenotype and secretory profile, thereby potentiating therapeutic efficacy. The application of tissue-mimetic 3D systems represents a promising strategy to amplify the regenerative capacity of AT-MSC-derived secretome and may serve as a crucial step toward the development of more effective regenerative therapies for chronic and non-healing wounds.

Lee et al. demonstrated that exosomes derived from AT-MSCs promote wound healing by enhancing the proliferation and migration of dermal fibroblasts and upregulating the expression of genes associated with tissue regeneration and collagen production. In animal models, the combination of exosomes with hyaluronic acid accelerated wound closure, increased re-epithelialization, and promoted type III collagen deposition, while in dermal filler models, it resulted in thicker tissue layers, enhanced vascularization, greater myofibroblast infiltration, and higher collagen fiber content. These findings indicate that exosomes favorably influence tissue regeneration and extracellular matrix remodeling, and their combination with hyaluronic acid may accelerate wound healing and improve the quality of regenerated skin, highlighting their potential as a promising tool in regenerative therapies [23].

Exosomes derived from human AT-MSCs play a crucial role in regulating skin repair processes and may support regenerative wound healing. Studies by Yu Fu et al. demonstrated that AT-MSC exosomes modulate keratinocyte and fibroblast activity, limiting scar formation and promoting the restoration of normal tissue architecture. Proliferative keratinocytes are particularly affected, exhibiting reprogramming capabilities via epithelial–mesenchymal plasticity mechanisms in response to exosome treatment. Importantly, AT-MSC-derived exosomes (AT-MSC-Exos) can reduce fibrosis by modulating the 14-3-3 zeta-YES-Hippo signaling pathway, offering new prospects for the development of effective therapies for scarless wound healing [12].

Li et al. demonstrated that AT-MSC-Exos play a crucial role in regulating wound healing by inhibiting the proliferation and migration of hypertrophic scar fibroblasts (HSFs) and reducing the expression of fibrosis markers (Col1, Col3, α-SMA) and TGF-β/Smad pathway-related signaling molecules (IL-17RA, p-Smad2/p-Smad3) while simultaneously increasing SIP1 levels. Furthermore, exosomal miR-192-5p, highly enriched in AT-MSC-Exos, attenuated hypertrophic scar formation by directly targeting IL-17RA. *In vivo*, treatment with AT-MSC-Exos accelerated wound closure and reduced collagen deposition, highlighting their therapeutic potential in preventing and treating pathological scarring [102].

Wang et al. demonstrated that AT-MSC-Exos play a pivotal role in wound healing by modulating the inflammatory response and promoting tissue regeneration. Their study showed that AT-MSC-Exos induced macrophage polarization toward the M2 phenotype, leading to a reduction in pro-inflammatory cytokines such as TNF-α and IL-6 and thereby creating a microenvironment conducive to tissue repair. Furthermore, IL-33 was identified as a key mediator of exosome activity, activating the Wnt/β-catenin signaling pathway in keratinocytes and enhancing cell proliferation, collagen deposition, and re-epithelialization. Experiments using IL33^−/− mice confirmed the critical role of IL-33 in this process, as its absence resulted in delayed wound closure and reduced M2 macrophage polarization. These findings indicate that the therapeutic potential of AT-MSC-Exos extends beyond the general regenerative properties of MSCs, involving precise modulation of cellular signaling and the wound microenvironment and highlighting their promise as a strategy for the treatment of chronic wounds, pathological scarring, and other skin injuries [103].

Ding et al. demonstrated that exosomes derived from adipose-derived stem cells accelerate wound healing by modulating the skin immune environment. *In vitro*, AT-MSC-Exos attenuated T-cell activation induced by PMA, suppressed pro-inflammatory cytokines (IL-2, IL-17A), restored Akt/PI3K signaling, and reduced apoptosis in human skin T cells. *In vivo*, AT-MSC-Exos decreased recruitment of dendritic epidermal T cells (DETCs) and reduced IL-17A levels at early wound sites, thereby preventing excessive inflammation. By limiting overactive immune responses and promoting a balanced inflammatory environment, AT-MSC-Exos facilitate more efficient wound closure and tissue repair [104].

Shao et al. demonstrated that adipose-derived stem cells overexpressing human growth hormone (HGH-AT-MSCs) significantly enhance the healing of burn wounds. *In vitro*, HGH-AT-MSCs promoted fibroblast proliferation, migration, and invasion, prolonged the G0/G1 phase of the cell cycle, and reduced apoptosis, effects that were dependent on ERK signaling. *In vivo*, HGH-AT-MSCs accelerated wound closure, enhanced re-epithelialization, increased collagen deposition, and reduced inflammation. Additionally, treated wounds showed decreased levels of pro-inflammatory cytokines (TNF-α, IL-1β, IL-6) and oxidative stress marker MDA, while antioxidant enzyme activities (SOD, CAT) were increased. These findings highlight the therapeutic potential of HGH-AT-MSCs in burn wound repair through fibroblast activation and ERK-mediated signaling modulation [105].

Gurney et al. demonstrated that engineered exosomes derived from adipose-derived stem cells (eXo 3) effectively promote wound healing. These exosomes express classical markers (CD9, CD63, CD81), display appropriate size, and maintain intact morphology. The exosomes were “tuned” in a bioreactor-based production system, allowing modification of their molecular cargo to enhance pro-regenerative activity, including promotion of collagen production, cell migration, and anti-inflammatory effects. *In vitro*, eXo 3 enhanced collagen production in fibroblasts and reduced pro-inflammatory cytokines (IL-6, IL-8, MCP-1), creating a regenerative microenvironment. They also accelerated fibroblast and keratinocyte migration, supporting re-epithelialization. *In vivo*, topical application in rodent excisional wound models increased cell proliferation and extracellular matrix deposition, leading to faster wound closure. Lyophilized exosomes retained bioactivity, demonstrating stability and therapeutic potential. These findings suggest a dual mechanism of action for eXo 3: promotion of tissue regeneration and modulation of inflammation, highlighting their promise as a cell-free therapy for wound repair [106].

Hyrije Ademi et al. investigated the effects of adipose-derived stem cell-conditioned medium (AT-MSC-CM) and TGF-β1 on human keratinocytes *in vitro*, focusing on proliferation, migration, apoptosis, and differentiation. AT-MSC-CM was found to contain high levels of growth factors and bioactive molecules, including HGF, FGFβ, VEGF, TIMP1/4, IL-8, PAI-1, uPA, and IGFBP-3. Treatment with AT-MSC-CM significantly enhanced keratinocyte proliferation compared to TGF-β1, promoted cell cycle progression (increased S phase DNA synthesis), and reduced apoptosis. Notably, AT-MSC-CM stimulated basal layer keratinocyte markers (DLL1, Jagged2 Notch ligands), supporting regenerative potential in epidermal repair. In contrast, TGF-β1 treatment primarily promoted late differentiation markers (CK10) but reduced basal keratinocyte proliferation. These findings suggest that AT-MSC-CM provides a strong trophic environment favoring basal keratinocyte proliferation, migration, and regenerative capacity, whereas TGF-β1 exerts more differentiation-oriented effects [107].

On the other hand, despite numerous publications confirming the efficacy of AT-MSC secretome in wound healing, the study by Zomer et al., which aimed to compare human dermal-derived MSCs and adipose-derived MSCs with their respective secretomes in terms of wound healing efficacy, demonstrated a limited therapeutic effect of the secretomes. Although secretomes possess therapeutic potential, the murine model showed intermediate outcomes between the treatment and control groups Specifically, the treatment groups received topical applications of either human dermal- or adipose-derived MSCs or their corresponding secretomes incorporated into a bilayer dermal scaffold, while the control group received the same scaffold without any biological component (empty scaffold control) or a vehicle solution (culture medium without active factors). The wounds treated with MSCs exhibited more developed granulation tissue, faster maturation of the neoepidermis, and better graft integration compared to the secretome-treated or control groups. The superior wound-healing properties of MSCs observed in Zomer’s study can be attributed to their secretion of paracrine factors in response to signals from the wound environment, which are absent in cultured MSCs. The limited efficacy of the secretome may be due to an insufficient dose, as the study did not compare different doses or single versus multiple applications [26]. The dose used had previously demonstrated effectiveness in *in vitro* wound healing (scratch cell) and angiogenesis studies [108]. However, given the lack of scientific consensus on optimal secretome dosing and application frequency, further optimization may be required for effective wound treatment in mice [26].

Lin et al. investigated the therapeutic potential of conditioned medium and extracellular vesicles derived from AT-MSCs on the healing of radiation-induced skin wounds. Using an *in vitro* model based on human dermal fibroblasts (HDFs) exposed to radiation, they demonstrated that both AT-MSC-CM and AT-MSC-EVs enhanced collagen synthesis, promoted the expression of genes associated with the extracellular matrix, and suppressed the expression of pro-inflammatory genes. Moreover, AT-MSC-EVs were observed to promote fibroblast proliferation. These findings suggest that AT-MSC-CM and AT-MSC-EVs may effectively accelerate skin regeneration following radiation-induced injury [109].

Interestingly, Urrata et al. aimed to characterize the secretome and exosomes derived from adipose-derived stem cell spheroids (SAT-MSCs) and to evaluate their potential in wound healing across various cell culture models. The study showed that SAT-MSC-derived exosomes were characterized by upregulated mRNA expression of NANOG and SOX2, markers associated with stemness, as well as miR-126 and miR-146a, which are implicated in angiogenic and osteogenic processes. Furthermore, the exosomes exhibited regenerative effects *in vitro*. These findings suggest that the AT-MSC secretome mediates paracrine signaling involved in maintenance of stemness, pro-angiogenic and pro-osteogenic differentiation, immune regulation, and tissue regeneration [110].

**Table 1:** Adipose-derived stem cells’ secretome potential in chronic wound treatment.

**AT-MSCs in Diabetes Chronic Wound Healing**			
**Paracrine Factors**	**Model**	**Therapeutic Effect**	**References**
Exosomes	mice normal wound healing model and a diabetic wound healing model.	Reduced inflammation. Promotion of angiogenesis, collagen deposition. Increased cellular proliferation and migration. Wound healing acceleration.	[24]
Exosomes	Rat skin fibroblasts and diabetic wound rat model	Enhanced the proliferation and migration of fibroblasts. Reduction of excessive myofibroblast differentiation and collagen deposition. Promotion of scarless healing of diabetic wounds *in vivo*.	[28]
Exosomes	HUVEC diabetes mellitus model *in vitro* and mouse normal wound healing model	Reduced oxidative stress and improved mitochondrial function *in vitro*. Increased proliferation and migration *in vitro*. Promotion of angiogenesis *in vitro* by increased ANG2, FILK1, and VEGF expression. Promotion of angiogenesis and improved wound healing *in vivo*.	[88]
Exosomes from miR-132-overexpressing cells	HUVEC diabetes mellitus model *in vitro* and diabetic wound mouse model	Improved skin flap survival and accelerated diabetic wound healing by attenuating local inflammation, promoting angiogenesis, and inducing M2 macrophage polarization via the NF-κB signaling pathway.	[89]
Exosomes	Diabetic wound mouse model	Increased wound healing by promoting contraction and re-epithelialization. Increased basal keratinocyte and dermal cell proliferation. Promotion of angiogenesis. Increased expression of VEGF, KGF, Col-I, and α-SMA. Production of collagen via TGF-β1/Smad3 signaling pathway	[90]
Exosomes	Diabetic rat skin fibroblasts transfected with miR-128-1-5p mimics and inhibitors and diabetic wound model *in vivo*	Increased cellular proliferation and migration. Suppressed TGF-β1 and α-SMA expression. Promotion of wound healing and attenuation of fibrosis and scar formation via the miR-128-1-5p/TGF-β1/Smad signaling pathway.	[91]
Exosomes from linc00511-overexpressing cells	Endothelial progenitor cells and rat diabetic foot ulcer model	Increased proliferation, migration, and angiogenesis. Diabetic foot ulcer healing improvement by suppressing PAQR3-induced Twist1 ubiquitin degradation to improve angiogenesis.	[92]
Exosomes from mmu_circ_0001052 overexpressing cells	HUVEC diabetes mellitus model and mouse diabetic foot ulcer model	Increased proliferation, migration, and angiogenesis. Improved wound healing by inflammation reduction and enhancement of granulation tissue formation. Mechanism via the miR-106a-5p/FGF4/p38MAPK pathway. Decreased apoptosis.	[93]
Conditioned medium (non-preconditioned DFX or preconditioned with DFX)	Diabetic wound/diabetic polyneuropathy mice model	Improved thermal and mechanical sensitivity. Restored intraepidermal nerve fiber density. Reduced neuron and Schwann cell apoptosis. Improved angiogenesis. Reduced chronic inflammation of peripheral nerves. Enhanced wound healing by wound closure, re-epithelization, and angiogenesis improvement.	[94]
Extracellular vesicles	Schwann cells and the diabetic peripheral neuropathy rat model	Enhanced proliferation of Schwan cells. Inhibited apoptosis. Promoted angiogenesis *in vivo*. Proposed mechanism via exosomal miR-130a-3p.	[98]
Exosomes	human immortalized keratinocyte cell line and diabetic wound mouse model	Induced autophagy (upregulation of NAMPT, CD46, VAMP7, VAMP3, EIF2S1). Enhanced epidermal cell proliferation and migration. Accelerated wound healing.	[100]
**AT-MSCs in Non-Diabetic Chronic Wound Healing**			
**Paracrine Factors**	**Model**	**Therapeutic Effect**	**References**
Exosomes	Mouse wound model	Promoted scarless wound healing through modulation of keratinocyte plasticity and keratinocyte–fibroblast interactions. Reduced fibrosis via the 14-3-3 zeta-YES-Hippo signaling pathway.	[12]
Exosomes	Human dermal fibroblasts, and *in vivo* wound healing mouse and porcine models	Increased proliferation and migration *in vitro*. Increased gene expression of collagen, α-SMA, FGF2, and elastin. Improved collagen deposition, wound closure, and re-epithelization when exosomes were combined with hyaluronic acid *in vivo*. Improved infiltration and differentiation of myoblast-induced extracellular matrix remodeling *in vivo*.	[23]
Conditioned medium	Mouse wound model	Wound closure and angiogenesis promotion. Efficacy limited when compared to the use of AT-MSCs as stem cell therapy.	[26]
Paracrine FactorsModel Therapeutic Effect Limitations Conditioned medium/Secretome from 3D tissue-mimetic hydrogel culture	*In vitro* keratinocyte and fibroblast wound-healing models	Enhanced secretion of proteins, antioxidants, and extracellular vesicles; increased proliferation, metabolism, and migration of keratinocytes and fibroblasts; improved regenerative response compared to 2D culture.	[73]
Exosomes	Schwann cells and sciatic nerve injury model	Decreased autophagy *in vitro* through miR-26b–mediated downregulation of Kpna2. Promoted myelin sheath regeneration *in vivo*.	[95]
Secretome from 3D electrospun scaffold culture	*In vitro* corneal fibroblast model and ex vivo rabbit corneal wound model	Increased levels of HGF and ICAM-1; accelerated fibroblast proliferation and migration; enhanced epithelialization and reduced scarring; decreased α-SMA expression, indicating inhibition of fibrosis.	[101]
Exosomes	Human skin fibroblast (HSF) and wound healing mouse model	Inhibited proliferation and migration, decreased expression of Col1, Col3, α-SMA, IL-17RA, and p-Smad2/p-Smad3 and increased the levels of SIP1 *in vitro*. Attenuated the excessive deposition of collagen, the trans-differentiation of fibroblasts to myofibroblasts, and the formation of hypertrophic scar by *in vitro* and *in vivo* experiments.	[102]
Exosomes	*In vitro* (keratinocytes, fibroblasts)	Promoted keratinocyte proliferation, collagen deposition, M2 macrophage polarization, anti-inflammatory effects, accelerated wound closure, enhanced collagen deposition, stimulated angiogenesis, promoted re-epithelialization via IL-33/Wnt/β-catenin pathway.	[103]
Exosomes	*In vitro* (HuT 78 human skin T cells) *In vivo* (C57 mouse full-thickness skin wound)	Attenuated T-cell activation (CD25), inhibited IL-2 and IL-17A expression, restored Akt/PI3K signaling, reduced apoptosis, reduced DETC recruitment, decreased IL-17A levels, limited early wound inflammation, promoted controlled healing.	[104]
HGH-overexpressing AT-MSCs (HGH-AT-MSCs)	*In vitro* (human dermal fibroblasts, HDF-a) *In vivo* (rat burn wound model)	Promoted fibroblast proliferation, migration, and invasion, reduced apoptosis, enhanced G0/G1 cell cycle progression and ERK-dependent signaling, accelerated wound closure, enhanced re-epithelialization, increased collagen deposition, reduced inflammation (TNF-α, IL-1β, IL-6), improved antioxidant status (increased SOD, CAT, decreased MDA).	[105]
Engineered AT-MSC exosomes (eXo 3)	*In vitro*: primary human fibroblasts and keratinocytes; *In vivo*: rodent excisional wound model	Increased collagen production, reduced pro-inflammatory cytokines (IL-6, IL-8, MCP-1), enhanced fibroblast and keratinocyte migration, promoted proliferation and ECM deposition, accelerated wound closure; stable after lyophilization; dual mechanism: regeneration + anti-inflammation.	[106]
Conditioned medium	*In vitro*: human keratinocytes	Stimulated basal keratinocyte proliferation and migration, enhanced S-phase DNA synthesis; reduced apoptosis, increased regenerative markers (DLL1, Jagged2, TGase3, loricrin); supported re-epithelialization.	[107]
Conditioned medium and extracellular vesicles	Radiation-induced skin injury model *in vitro* (human dermal fibroblasts)	Enhanced extracellular matrix deposition. Increased expression of COL1A1, COL1A2, and COL3A1. Decreased IL-6 cytokine. Promoted proliferation in the extracellular vesicles group.	[109]
Exosomes from spheroids of adipose stem cells	Endothelial cells Fibroblasts osteoblasts	Improved wound healing. Exosomes from spheroids of adipose stem cells are characterized by upregulation of NANOG and SOX2, as well as miR126 and miR146a.	[110]

### 4.2. Skin Aging

As the body’s primary protective barrier, the skin undergoes continuous aging. This process is influenced by both intrinsic and extrinsic factors. Photoaging, defined as excessive exposure to ultraviolet (UV) radiation, is one of the primary extrinsic factors contributing to skin aging [111]. Photoaging affects the morphological and physiological characteristics of the skin, contributing to the formation of wrinkles and a reduction in skin elasticity [112]. This is associated with a slowdown in cell proliferation, decreased production of collagen types I and III, and elastin. Additionally, UV radiation reduces the synthesis of procollagens and activates signaling pathways such as mitogen-activated protein kinase (MAPK) and activator protein 1 (AP-1). It also leads to the accumulation of reactive oxygen species (ROS), ultimately resulting in cellular apoptosis [113,114,115,116].

Recent studies have shown that the AT-MSC secretome can promote skin rejuvenation and counteract aging processes [13]. Guo et al. demonstrated in an *in vitro* model that the AT-MSC secretome can slightly improve fibroblast proliferation and reduce cellular senescence. Moreover, increased expression of collagen I and II as well as elastin and TIMP-1 were observed, which can affect deposition and remodeling of the extracellular matrix. The photoprotective effects were attributed to the presence of platelet-derived growth factor-AA (PDGF-AA) within the AT-MSC secretome [116]. Cho et al. also showed that fibroblasts treated with TGF-ß1-treated AT-MSC-CM increased expression of collagen I. Moreover, fibroblasts were characterized with higher proliferation and migration potential, which can affect the wound healing process [117].

The regenerative mechanism of the AT-MSC secretome is associated with the activation of the Wnt/β-catenin signaling pathway. Upregulation of Wnt3a and β-catenin promotes increased levels of TGF-β2, a key factor in type I procollagen synthesis. Xu et al. demonstrated that AT-MSC-CM can increase fibroblast proliferation and the expression of skin regeneration-related proteins through activation of the Wnt/β-catenin signaling pathway, thereby promoting the renewal of photoaged skin [118]. In addition, Putri et al. demonstrated that a four-week treatment with the AT-MSC secretome in male Wistar rats improved the condition of photoaged skin through paracrine mechanisms, modulation of MMP-1 and TIMP-1 expression, as well as increased epidermal thickness and collagen density in the dermis [119].

Research on the application of the AT-MSC secretome in photoprotection remains significantly limited. A detailed understanding of the secretome’s mechanisms in the anti-aging process could provide a foundation for its broader use. Studies conducted by Kim et al. demonstrated that the secretome may serve as an effective active ingredient in cosmetic products applied via spray using an airbrush. These formulations contained up to 5% secretome, and their use resulted in a reduction in wrinkle visibility and an improvement in skin hydration levels [120]. In another study, researchers used the secretome in the form of a cream, achieving a concentration of 1% through three-dimensional (3D) culture. The results suggest that the use of this formulation may more effectively stimulate collagen synthesis and improve skin elasticity compared to the control group, thereby contributing to the deceleration of aging processes [120].

Importantly, in a clinical study conducted by Svolacchia et al., involving 72 women without pathological inflammation of the dermis or epidermis—except for age-related and photoaging-induced changes—significant skin improvement was observed after 30 days of facial microinjection treatment with an AT-MSC-derived exosome suspension. The retrospective study demonstrated that this method for treating facial wrinkles and tissue folds is safe, effective, and innovative. The therapy exerts regenerative effects, representing a potent alternative to conventional anti-aging procedures and treatments for inflammatory conditions, as it lacks pro-inflammatory components and may reduce the presence of mesenchymal cells as well as multiple inflammatory cytokines [27].

These results are particularly promising in the context of protecting the skin against the harmful effects of UV radiation and other environmental factors, potentially opening new perspectives for the application of the AT-MSC secretome in advanced dermocosmetic formulations with protective and anti-aging properties (Table 2).

### 4.3. Alopecia

Alopecia is a medical condition characterized by the loss of hair from the scalp and/or other parts of the body. This condition may be triggered by a variety of factors, including genetic predisposition and environmental influences [122]. Androgenetic alopecia is the most common type of hair loss disorder worldwide, affecting both men and women. The use of AT-MSC-derived secretome represents a form of stem cell therapy aimed at the restoration of skin structures, including hair follicles [123,124].

At the molecular level, the pathogenesis of the androgenetic alopecia condition involves the binding of dihydrotestosterone (DHT) to the androgen receptor [98]. Tang et al., using human hair follicles, dermal papilla cells *in vitro*, and *in vivo* animal models, demonstrated that AT-MSC exosomes stimulated hair growth by counteracting the inhibitory effects of DHT. Furthermore, they observed an increased level of Ser9-phosphorylated glycogen synthase kinase 3β (GSK-3β) and enhanced translocation of β-catenin, a process that could be blocked by dickkopf-related protein 1 (DKK1), an inhibitor of the Wnt/β-catenin signaling pathway [125]. Fu et al. also demonstrated intercellular communication mediated by human AT-MSC exosomes through the Wnt/β-catenin signaling pathway and improvement in hair regeneration in androgenic alopecia in a mouse model. By activating the Wnt/β-catenin pathway via CDC42, AT-MSC exosomes may potentially inhibit the expression of GSK-3β, thereby counteracting the suppressive effects of DHT, promoting cell proliferation, migration, and enhancing hair growth [126]. Moreover, Li et al. demonstrated that AT-MSC exosomes promote proliferation and migration but reduce apoptosis of dermal papilla cells. The researchers also showed improved hair growth (more hair follicles and thicker dermis) *in vivo* [127]. Similarly, Park Byung-Soon et al. demonstrated that AT-MSC-CM increased the proliferation of keratinocytes and follicle dermal papilla cells. Moreover, the secretome of AT-MSCs stimulated hair growth in female C3H/HeN mice by induction of the anagen phase. In addition, hypoxic conditions further enhanced this effect. This enhancement was associated with an increased secretion of growth factors such as IGFBP-1, IGFBP-2, M-CSF, M-CSF R, PDGF R-β, and VEGF, all present in the conditioned medium [128]. Furthermore, Choi et al. demonstrated that AT-MSC-CM treated with heparin-binding epidermal growth factor-like growth factor (HB-EGF), when injected into the dorsal skin of mice, stimulated *in vivo* hair growth. This effect was also associated with increased expression of growth factors [129]. It has also been shown that AT-MSC-CM can increase hair number and improve hair regeneration in patients with alopecia [130].

The presented studies demonstrated a positive effect of AT-MSC secretome on hair repair and alopecia treatment, indicating its potential for use in clinical practice (Table 3).

### 4.4. Immune-Related Skin Diseases

AT-MSC secretome has remarkable immunomodulatory properties due to secretion of anti- and pro-inflammatory factors and thus can be used for treatment of immune-related skin diseases to alleviate inflammation; its efficiency has already been documented in *in vitro* and *in vivo* studies [7,22]. It has been shown that AT-MSC-CM can decrease pro-inflammatory expression of COX2 and MMP3 markers by human dermal fibroblasts (hDFs) previously induced with IL-1 *in vitro*. However, the decrease in COX2 gene expression was more pronounced than that of MMP3. Moreover, hDF proliferation was increased after AT-MSC-CM application, but it is worth noting that higher dose of AT-MSC-CM did not enhance that effect. Similarly, expression of the MMP3 and COX2 genes was decreased in human epidermal keratinocytes *in vitro*; however, the enhanced proliferation effect was AT-MSC-CM dose-dependent. The same group also showed that AT-MSC-CM could prevent strong inflammatory reaction in an *in vivo* model and suppress skin redness and thickness [131].

AT-MSC secretome may be used for the treatment of chronic autoimmune diseases such as psoriasis. Kim et al. showed that AT-MSC exosomes can reduce inflammation and oxidative stress in HaCaT cells pre-treated with psoriasis serum-derived exosomes from patients. AT-MSC exosome treatment on psoriasis-induced keratinocytes *in vitro* significantly decreased proinflammatory expression of cytokines such as IL-1β, IL-6, and TNF-α, reduced oxidative stress-related gene expression (NOX2 and NOX4), as well as regulated autophagy by increasing expression of ATG5, P62, Beclin1, and LC3B factors [132]. In the case report by Mahmood et al., a topical application of 1–2 mL of concentrated AT-MSC-CM once a day for 4 weeks was used to treat scalp psoriasis. The results showed improvement in psoriatic plaques, elimination of silver scales, and restoration of natural skin color. Moreover, no side effects were reported by the patient, and after 3 years of follow up, the patient remained disease-free [133]. Similarly, in 2019, the same research group showed improvement in the regression of patients’ psoriasis vulgaris after topical application of concentrated AT-MSC-CM once a day for 4 weeks [134].

AT-MSC secretome may also be used for the treatment of atopic dermatitis (AD). Shin et al. showed that subcutaneous injection of exosomes derived from AT-MSCs could notably reduce pro-inflammatory cytokine (IL-4, IL-5, IL-13, TNF-α, IFN-γ, IL-17, and TSLP) concentrations in a dose-dependent manner and decreased the level of IgE in serum. Moreover, AT-MSC exosomes improved skin barrier restoration by decreasing trans-epidermal water loss, promoting stratum corneum hydration by significant induction of ceramide production [135]. Another study, by Pang et al., showed that topical application of membrane-free stem cell extract (MFSCE) from AT-MSCs—prepared by disruption of membrane with ultrasonication, followed by centrifugation and filtration—can inhibit erythema, dry skin, edema, erosion, and lichenification in an AD mouse model. MFSCE treatment *in vivo* improved skin condition (epidermal thickness, infiltration of mast cells) and reduced serum IgE concentration as well as pro-inflammatory cytokines (IL-4, IL-10 IFN-γ, TNF-α) and chemokine (TARC) involved in the regulation of Th1 and Th2 related inflammatory response [136]. In addition, Cho et al. showed that AT-MSC exosome injection can reduce the expression of cytokines such as IL-4, IL-23, IL-31, and TNF-α, as well as the IgE level in an *in vivo* AD mice model. Treatment with AT-MSC exosomes helped relieve AD symptoms by decreasing eosinophils in blood, reducing CD86+ and CD206+ cells, and significantly reducing infiltrated mast cells in skin lesions [137]. In addition, an *in vitro* study by Roh et al. demonstrated the efficiency of AT-MSC exosomes in an AD-like triple-cell model (HaCaT, HDF, and HMC-1 cell lines) treatment. AT-MSC exosomes suppressed expression of proinflammatory cytokines (IL-6, IL-1β, and IL-1α) and increased expression of skin barrier genes (FLG, LOR). The same effect was observed at the level of protein secretion [138].

The described studies have shown that AT-MSC-secretome has significant therapeutic potential in treating inflammatory skin conditions. The ability of AT-MSC secretomes to modulate the inflammatory response, reduce inflammation, and promote tissue regeneration highlights their potential for use in stem cell-free therapeutic strategy (Table 4).

## 5. A Preconditioning Approach to Modulate the Cellular Secretome

AT-MSC stimulation with physical or biochemical inducers may help increase the regenerative potential of AT-MSCs for regenerative medicine purposes in both stem cell-based therapy and stem cell-free therapy. Moreover, genetic modifications of MSCs are considered to increase the therapeutic potential of the MSC secretome. Below, we describe possible preconditioning approaches based on physical stimulation for enhancing the AT-MSC secretome’s regenerative potential. A preconditioning approach based on physical stimulation (e.g., electromagnetic fields, electricity, hypoxia, mechanical pressure) is potentially safer and more cost-effective than using biochemical factors such as cytokines or growth factors. However, the biochemical preconditioning strategy is more extensively studied and may allow for more targeted modulation of cellular behavior by engaging defined molecular pathways [11,29,139,140,141].

### 5.1. Low-Frequency Electromagnetic Fields

Low-frequency electromagnetic fields (LF-EMFs) are non-ionizing electromagnetic fields characterized by low energy (radiation). LF-EMFs can influence the biological processes of MSC, such as proliferation or differentiation. They can modulate MSC secretome, important for skin regeneration by increasing the concentration of secreted biomolecules with re-epithelizing, pro-angiogenic, and immunomodulatory properties [7,141].

One of the most important proteins for the skin regeneration process, especially in the wound healing process, is FGF-2. This growth factor is involved in cell migration, proliferation, neoangiogenesis, and synthesis and deposition of components of the extracellular matrix. However, the local application of FGF-2 for wound healing faces a significant limitation related to low biological stability due to the proteolytic wound environment [7,49,142,143,144]. Trzyna et al. showed that an LF-EMF (50 Hz; 1.5 mT) increased FGF-2 secretion by AT-MSCs *in vitro* threefold after 48 h of continuous stimulation [145]. Notably, studies showed that LF- EMFs may increase FGF-2 secretion by endothelial cells *in vitro*, improving angiogenesis and the wound healing process in *in vivo* models [144,146]. Moreover, Trzyna et al. showed a slight increase in VEGF and HGF secretion by AT-MSCs after 48 h of LF-EMF treatment (50 Hz; 1.5 mT) [145]. Interestingly, Marędziak et al. showed that a static magnetic field (0 Hz; 0.5 T) may increase VEGF concentration in equine AT-MSC microvesicles, which may be promising for veterinary regenerative medicine [147].

Overall, LF-EMFs can be a promising tool for enhancing AT-MSC secretome activity for skin regeneration processes; however, it is important to note here that the effect of an LF-EMF may be dependent on its parameters, such as frequency, magnetic induction, and exposure time. Moreover, increased concentrations of growth factors promoting skin regeneration—especially naturally encapsulated in vesicles—secreted by AT-MSCs after LF-EMF exposure may be a promising approach for clinical applications in dermatology. However, there are a limited number of studies about the AT-MSC secretome profile after LF-EMF treatment focused on the skin regeneration aspect, and further research is required.

### 5.2. Hypoxia

Hypoxic preconditioning is a method involving the exposure of MSCs to hypoxic conditions, with the aim of enhancing their therapeutic and regenerative potential through modifications in the composition of the secretome. A low-oxygen environment induces changes in the expression of genes involved in tissue repair and regeneration mechanisms, which is why it has become the subject of research [148]. The AT-MSC secretome, due to its composition and properties, supports the processes involved in skin regeneration, for example angiogenesis, as well as tissue repair by improving wound healing. The properties of the secretome and consequently, the benefits it provides—depend on the culture conditions. Therefore, by manipulating various culture conditions of AT-MSCs, it is possible to obtain secretomes with different compositions and properties, which in turn affect their function. Therefore, the AT-MSC secretome has emerged as a promising tool for stem cell-free skin therapy [149,150].

Studies in a preclinical mouse model have shown that the application of secretome obtained under both hypoxic and normoxic conditions, as well as serum-free conditions, had a positive impact on regenerative properties and accelerated wound healing. Furthermore, the AT-MSC secretome contributes to an increase in blood vessel density and the presence of pericytes at the site of injury [150]. Moreover, in the study by Hermann et al. it was demonstrated that the use of platelet lysate (PL) in AT-MSC culture, combined with hypoxic conditions and epidermal growth factor (EGF) treatment, enhances the wound healing process by stimulating keratinocyte migration and viability, as well as by increasing the secretion of growth factors such as VEGF, EGFR, FGF-4, M-CSF, and SCF [149].

In the study by Kalinina et al., cell secretome from AT-MSCs cultured under normoxic conditions (48 h incubation, 37 °C, 5% CO_2_, 21% O_2_) and hypoxic conditions (48 h incubation, 37 °C, 5% CO_2_, 1% O_2_) were compared. Hypoxia led to an increase in HIF-1α levels and the secretion of six proteins (including proteins involved in the angiogenesis process, such as EGF-like repeats and discoidin I-like domains 3 (EDIL), adrenomedullin, and ribonuclease 4 of the RNase A family), which were absent in the secretomes of cells incubated under normoxic conditions. Additionally, seven proteins, including CRTAP—a protein associated with the process of osteogenesis—were not detected in the hypoxic samples [151]. Moreover, it has also been demonstrated that the AT-MSC secretome cultured under hypoxic conditions, when combined with a collagen scaffold, supports angiogenesis *in vivo* more effectively compared to the secretome obtained under normoxic conditions. Additionally, hypoxia induces the production of a secretome enriched with pro-angiogenic factors, such as adiponectin, bFGF, GRO, GRO-α, ENA78, and ICAM1-3 [152].

Hypoxia is also a stress condition that is important in terms of mimicking the post-transplant environment. In the study by Pinheiro-Machado et al., the secretomes of rat and human pancreatic islets were compared under different conditions—both alone and co-cultured with AT-MSCs—that simulated transplant-associated environments. These included the presence of cytokines, elevated glucose levels, hypoxia, normoxia, and a combination of high glucose and hypoxia. The results showed that hypoxia alone, as well as hypoxia combined with high glucose levels, led to an increased secretion of collagen type I alpha 1 and pro-angiogenic factors such as VEGF, PDGF, and bFGF, as well as the anti-inflammatory cytokine IL-10 [153].

A similar culture condition was also examined in the study by Pinheiro-Machado et al., where the composition and functions of the AT-MSC secretome from perirenal fat in humans and rats were investigated. In this case, hypoxia also led to an increased secretion of proteins, including proangiogenic factors (VEGF), proteins associated with the ECM, immune-related proteins (complement proteins and interleukins), type IV collagens, MMP-2 and MMP-9 proteins, IGFBP3, IGFBP4, and annexin A1. Hypoxia combined with a high glucose concentration also caused an increase in VEGF, various types of collagen, and beta-galactosidase 1 [154]. Similarly, in the study by Frommer et al., hypoxia (and in this case also, the action of TGF-β1 and IL1-β) led to an increased expression of VEGFA, FGF2, IL6, and factors related to hemostasis such as SERPINE1 and PLAUR, as well as various components of the extracellular matrix. All these factors are associated with wound healing, inflammation, and skin fibrosis [155].

It has been also demonstrated that combining three-dimensional culture (which allows for a better replication of the *in vivo* environment, resulting in higher levels of TGF-β1 and VEGF in AT-MSC-CM) with hypoxia (which increases the secretion of factors such as VEGF, HGF, and bFGF) creates optimal conditions for obtaining an AT-MSC secretome with enhanced therapeutic effects, particularly in the treatment of hypertrophic scars [156].

The studies described above confirm that hypoxic culture conditions of AT-MSCs significantly enhance their therapeutic potential by modifying the secretome—particularly in the context of wound healing, angiogenesis, and immunomodulation.

## 6. Overview of Available Clinical Studies

Adipose-derived stem cells exhibit considerable potential for clinical application due to their pro-regenerative properties, and the interest in their use has been increasing annually. Currently, the global Clinical Trials database maintained by the National Library of Medicine (NLM) at the National Institutes of Health (NIH)—clinicaltrials.gov [157] lists 433 clinical trials investigating AT-MSCs across various domains, including vascular, hematological, respiratory, reconstructive, skeletal, ophthalmological, and neurological applications. Additionally, numerous studies explore the use of AT-MSCs for skin regeneration, addressing conditions such as chronic wounds, ulcers, burns, scars, psoriasis, atopic dermatitis, alopecia, fistulas, and skin aging/rejuvenation. However, the majority of these studies focus on interventions involving injection, infusion, or local administration of AT-MSCs, while only a few consider the use of AT-MSC secretome alone. These studies include applications for androgenetic alopecia and skin aging/rejuvenation (Table 5).

Three studies have investigated the treatment of androgenetic alopecia and its treatment with AT-MSC-CM, and their results are available [158,159,160]. The interventional, randomized single-blind clinical trial NCT06066827 determined that the best therapeutic effect was achieved when AT-MSC-CM was combined with minoxidil, a commonly used agent for promoting hair growth. The authors hypothesize that this effect might be attributed to a synergic mechanism wherein minoxidil stimulates the production of VEGF, while AT-MSC secretome is naturally rich in VEGF. An increased concentration of VEGF enhances perifollicular angiogenesis, thereby augmenting the size of hair shafts and follicles. Furthermore, the study showed that synergic use of AT-MSC-CM and minoxidil may mitigate the side effects of minoxidil and prevent hair shedding after 8 weeks [158]. Similarly, a randomized double-blind clinical trial, number NCT05296863, reported patient satisfaction and minimal side effects with the combined use of AT-MSC-CM and 5% minoxidil. However, no significant difference was observed between the study groups, as both received the combined treatment, albeit with one receiving a concentrated form of CM and the other a non-concentrated form [159]. Furthermore, the observational retrospective study NCT05129800 evaluated and compared the therapeutic efficacy in the treatment of androgenetic alopecia using platelet-rich plasma (PRP) from two different companies and mesotherapy with ampules containing AT-MSC-CM and a mixture of growth factors (MZT1 and MZT2) from two distinct companies. The findings revealed variations in the effects of AT-MSC-CM depending on the supplier. Patients treated with MZT1 exhibited a statistically significant improvement in hair count and hair thickness in both the vertex and frontal areas, as well as increased hair density in the frontal area. In contrast, patients receiving MZT2 showed improvement only in hair thickness in the vertex area. The authors suggest that the observed differences between MZT1 and MZT2 may be attributed to variations in group sizes, gender distribution, and average age among participants receiving AT-MSC-CM. Additionally, discrepancies in the concentration of recombinant growth factors and AT-MSC-CM, the injection method (direct versus injector device), the dosage applied per cm^2^, and the experience of the individual administering the injection may also contribute to these differences [160]. Nevertheless, based on the aforementioned studies, it can be concluded that the use of AT-MSC-CM may serve as an effective intervention for the treatment of androgenetic alopecia.

Moreover, the single-blind randomized clinical trial NCT05508191 investigated the efficacy of concentrated AT-MSC-CM in conjunction with either fractional CO_2_ laser (FL) or fractional microneedle (MN) procedures for facial skin rejuvenation. The findings indicated that both methods effectively enhanced the total dermoscopy photoaging scale, including improvements in fine wrinkles and hyperpigmented macules, with no significant clinical differences between the two tested groups. The authors note, however, that while both methods are effective, the selection of a specific technique may be influenced by patient preference and anticipated comfort, as well as the potential to mitigate side effects such as skin redness [161].

It is worth noting that four more studies with human subjects using AT-MSC secretome were conducted. Those studies were not registered in the ClinicalTrials.gov database; therefore, the results were discussed in the “Skin-aging” and “Alopecia” subsections in this review paper [27,120,121,130].

## 7. Limitations of the Reviewed Studies

While the therapeutic potential of AT-MSC-derived secretome, exosomes, and conditioned media in chronic wound healing, skin regeneration, hair growth, and inflammatory skin disorders is promising, several significant limitations of the current studies must be acknowledged. The majority of evidence originates from preclinical studies, including animal models (mice, rats) and *in vitro* cultures, such as dermal fibroblasts, keratinocytes, endothelial cells, and immortalized cell lines. These models do not fully replicate the complexity of human chronic wounds, skin aging, alopecia, or inflammatory skin diseases, which limits the translational potential of the findings. Many studies focus on specific molecular pathways, such as Wnt/β-catenin, TGF-β/Smad, or NF-κB, without assessing broader networks or interactions between different cell types, potentially overlooking important mechanisms involved in tissue regeneration.

Another limitation is the short duration of most preclinical studies, which hampers the evaluation of the long-term stability of wound healing, hair regrowth, and skin regeneration, and the safety of the interventions. Additionally, many studies assess only molecular and histological markers, with limited functional evaluation of the tissues, which may not fully reflect therapeutic efficacy in clinical settings. Some studies involve genetic or pharmacological modifications of MSCs, such as miRNA overexpression or HGH stimulation, raising additional questions regarding clinical safety and regulatory considerations. Technical limitations, including the use of 2D versus 3D culture systems and selective cell types, may also affect the composition and regenerative potential of secretome or exosomes.

In recent years, initial clinical studies investigating AT-MSC-CM have emerged, demonstrating promising results in the treatment of androgenetic alopecia and skin rejuvenation. However, the number of clinical studies remains limited, underscoring the need for further well-designed clinical trials involving larger patient populations and longer follow-up periods to comprehensively assess the safety, efficacy, and translational potential of AT-MSC-based therapies.

In summary, these limitations highlight the necessity for continued preclinical and clinical research to fully elucidate the therapeutic potential of AT-MSC-derived secretome in regenerative and cosmetic applications.

## 8. Conclusions

The adipose-derived stem cell secretome shows significant potential for skin regeneration and offers a cell-free alternative to conventional stem cell-based therapies. The unique properties of the AT-MSC secretome result from the presence of numerous active biomolecules such as cytokines, proteins, growth factors, RNAs, and exosomes with immunomodulatory, re-epithelializing, proangiogenic, neurotrophic, anti-fibrotic, and antiapoptotic activity. The application of AT-MSC secretome may represent a more effective alternative treatment than the administration of whole cells. Additionally, the advantages of stem cell-free therapies include reduced immunogenicity, easier storage and handling, and potentially lower production costs compared to stem cell-based therapies. The studies discussed in this review showed that the AT-MSC secretome can be an effective tool for skin regeneration in wound healing, skin rejuvenation, alopecia, and immune-related skin diseases. Moreover, the use of a preconditioning strategy based on the stimulation of AT-MSCs with physical factors, such as low-frequency electromagnetic fields or hypoxia, may increase the pro-regenerative properties of the AT-MSC secretome. Nevertheless, further research is required to understand the specific role of AT-MSC secretome in skin regeneration. Moreover, challenges remain in standardizing secretome production, optimizing delivery methods, and ensuring long-term efficacy and safety of the stem cell-free therapy approach for skin regeneration purposes. Continued advances in research and development may lead to secretome-based treatments becoming safe, effective, and accessible alternatives to traditional stem cell therapies in dermatology and regenerative medicine.

## Figures and Tables

**Table 2:** Adipose-derived stem cell secretome potential in skin aging/skin rejuvenation.

Paracrine Factors	Model	Therapeutic Effect	References
Exosomes	Clinical use (case report/clinical study)	A significant improvement in skin parameters was observed following the application of this method.	[27]
Conditioned medium	Human dermal fibroblasts *in vitro*	Promoted ECM remodeling and collagen synthesis; photoprotection.	[116]
TGF-ß1-treated conditioned medium	Human skin fibroblasts *in vitro*	Increased expression of I collagen and MMP-1. Proliferation and migration promotion.	[117]
Conditioned medium	Human dermal fibroblasts *in vitro*	Increased proliferation and the expression of skin regeneration-related proteins through activation of the Wnt/β-catenin signaling pathway.	[118]
AT-MSC secretome	Photoaging rat model *in vivo*	Increased epidermal and dermal layer thickness and dermal collagen density. Increased MMP-1 and TIMP-1 expression.	[119]
Conditioned medium	Clinical use (human skin—cosmetic application)	Reduced wrinkle visibility, improved hydration.	[120]
Conditioned medium	Clinical use (human skin—cosmetic application)	Increased collagen production, skin repair, and dermal density.	[121]

**Table 3:** Adipose-derived stem cells’ secretome potential in alopecia treatment.

Paracrine Factors	Model	Therapeutic Effect	References
Exosomes	Human hair follicles, alopecia mice *in vivo*	Hair growth improvement.	[125]
Exosomes	Alopecia mice *in vivo*	Increased cell proliferation and migration. Hair growth improvement. Inhibited GSK-3β. Activated Wnt/β-catenin pathway.	[126]
Exosomes	Dermal papilla cells, alopecia mice *in vivo*	Increased proliferation and migration and reduced apoptosis of dermal papilla cells. Hair growth improvement.	[127]
Conditioned medium	Alopecia mice *in vivo*	Promoted hair growth by inducing the anagen phase. Enhanced proliferation of hair follicle cells and keratinocytes.	[128]
Conditioned medium	Alopecia mice *in vivo*	Stimulated *in vivo* hair growth; increased expression of growth factors	[129]
Conditioned medium	Clinical use	Increased hair number, improved hair regeneration.	[130]

**Table 4:** Adipose-derived stem cells’ secretome potential in immune-related skin pathologies.

Paracrine Factors	Model	Therapeutic Effect	References
Conditioned medium	human dermal fibroblast and human epidermal keratinocyte inflammatory model *in vitro*	Decreased MMP3 and COX2 gene expression. Enhanced proliferation of cells.	[131]
Conditioned medium	inflammation mouse model *in vivo*	Reduced inflammatory skin reaction to PMA.	[131]
Exosomes	*in vitro* model of psoriasis	Decreased expression of proinflammatory cytokines IL-1β, IL-6, and TNF-α, as well as oxidative stress-related NOX2 and NOX4. Autophagy process regulation by increasing ATG5, P62, Beclin1, and LC3B protein expression.	[132]
Conditioned medium	clinical use (case study)	Improvement in psoriatic plaques, elimination of silver scales, restoration of natural skin color.	[133]
Conditioned medium	clinical use (case study)	Improvement in psoriatic plaques, elimination of silver scales. Reduction of PSSI score to 0 from 28.	[134]
Exosomes	atopic dermatitis mouse model *in vivo*	Improvement of skin barrier restoration. Reduction of serum IL-4, IL-5, IL-13, TNF-α, IFN-γ, IL-17, TSLP, and IgE.	[135]
Membrane-free stem cell extract	atopic dermatitis mouse model *in vivo*	Improvement of skin condition. Reduction of serum IgE, IL-4, IL-10 IFN-γ, TNF-α, TARC.	[136]
Exosomes	atopic dermatitis mouse model *in vivo*	Alleviation of atopic dermatitis. Reduction of IL-4, IL-23, IL-31, TNF-α, IgE.	[137]
Exosomes	atopic dermatitis triple-cell model *in vitro*	Reduction of IL-6, IL-1β, and IL-1α gene expression. Reduction of IL-4, IL-6, and IL-1β protein expression. Increased FLG, LOR gene expression. Increased FLG protein expression.	[138]

**Table 5:** Clinical trials of adipose-derived stem cells’ secretome for cell-free-based therapies [157]. AT-MSC-CM—Adipose-derived stem cells—conditioned media.

NCT No.	Condition	Number Enrolled	Intervention/Treatment	Results	Reference
NCT06066827	Androgenetic Alopecia	60	Three groups: Injection of AT-MSC secretome concentrate; topical use of minoxidil; injection of AT-MSC secretome concentrate combined with topical use of minoxidil.	Statistically significant improvement in hair growth parameters in all three groups. Best results were observed in the combination group (AT-MSC-CM with Minoxidil). Minimal side effects were reported.	[158]
NCT05296863	Androgenetic Alopecia	37	Two groups: Intradermal injection of non-concentrated AT-MSC-CM combined with topical application of 5% Mixidil daily; intradermal injection of concentrated AT-MSC-CM combined with topical application of 5% Mixidil daily.	Significant increase in hair growth in both groups. Minimal side effects were reported.	[159]
NCT05129800	Androgenetic Alopecia	72	Four groups: Injection of platelet-rich plasma prepared using a tube from company 1 (PRP1); injection of platelet-rich plasma prepared using a tube from company 2 (PRP2); mesotherapy with ampule containing AT-MSC-CM and mixture of recombinant growth factors from company 1 (MZT1); mesotherapy with ampule containing AT-MSC-CM and mixture of recombinant growth factors from company 2 (MZT2).	Significant improvement in hair growth parameters in groups MZT1 and PRP2. Significant improvement in vertex hair density in MZT2 group.	[160]
NCT05508191	Skin Aging/Rejuvenation	30	Two groups: four-fold concentrate of AT-MSC-CM and fractional CO_2_ Laser; four-fold concentrate of AT-MSC-CM and fractional microneedle.	Significant improvements in the total dermoscopy photoaging scale, improvements in fine wrinkles and hyperpigmented macules in both groups.	[161]

## Data Availability

No new data were generated or analyzed in support of this research. All data discussed in this review are available in the cited literature.
